# Identification of Candidate Genes Associated with Growth Traits in *Procambarus clarkii* Using Whole-Genome Resequencing

**DOI:** 10.3390/ijms27177738

**Published:** 2026-08-29

**Authors:** Jian Li, Pingping Hu, Huiling Zhang, Yiming Luo, Xingfei Huang, Dongwu Wang, Jinlong Li, Zhiming Wang, Yude Wang, Shaojun Liu

**Affiliations:** 1 Engineering Research Center of Polyploid Fish Reproduction and Breeding of the State Education Ministry, Hunan Normal University, Changsha 410081, China; 2 Yuelushan Laboratory, Changsha 410128, China; 3 Department of Laboratory Animal Science, University of South China, Hengyang 421001, China; 4 Changsha Agricultural Science Research Institute, Changsha 410026, China

**Keywords:** *Procambarus clarkii*, growth, whole-genome resequence, signatures of selection, GWAS

## Abstract

Growth is a critical economic trait in all aquaculture industries. To address issues such as germplasm degradation, a comprehensive understanding of the growth and development mechanisms, along with genetic improvement strategies, for *Procambarus clarkii* (*P. clarkii*) is urgently required. In this study, we performed whole-genome resequencing on 89 individuals from five cultured stocks to investigate growth traits (body length) and identified a total of 46,919,297 high-quality single nucleotide polymorphisms (SNPs). Based on these SNPs, we conducted principal component analysis (PCA), phylogenetic analysis, and population genetic structure analysis. Furthermore, we performed selective sweep analysis (using FST, Pi, and XP-CLR) and a genome-wide association study (GWAS) to identify genetic variants associated with growth traits. The results revealed significant genetic differentiation among the five cultured stocks, with the Ma’anshan cultured stock exhibiting the fastest linkage disequilibrium (LD) decay. Additionally, long-term aquaculture in different geographical regions resulted in distinct genetic differences among cultured stocks. Through selective sweep analysis, the intersection of FST, Pi, and XP-CLR across the five populations yielded several growth-related candidate genes: Nephrin, Somatostatin, zinc finger protein 154, and yeti. Subsequent the GWAS identified two candidate genes associated with growth traits: Cullin-associated and neddylation-dissociated protein 1 (CAND1) and Baculoviral IAP repeat-containing protein 8 (BIRC8). These genes are presumed to play pivotal roles in the growth and development of *P. clarkii*. Overall, our findings provide new insights into the genetic mechanisms underlying growth and development in *P. clarkii*, and these identified genes serve as promising candidates for further functional studies and genetic improvement of this species.

## 1. Introduction

*Procambarus clarkii*, native to northeastern Mexico and the south-central United States [1], is one of the most invasive species in the world [2,3] and one of the most widely distributed freshwater crayfish [4]. In the 1930s, *P. clarkii* was introduced to China and quickly became one of the important aquatic economic species in the country [5]. According to the “China Crayfish Industry Development Report (2025)”, the total aquaculture area has reached 20,333 square kilometers, with an annual production of 3.4476 million tons [6]. Due to its high nutritional value, it has rapidly become a species of significant economic importance [7]. Therefore, improving the quality of *P. clarkii* through genetic improvement and selective breeding is of great significance, particularly with regard to further research on the trait of body size.

In aquaculture, growth, development, and body size are important research targets that directly affect yield and economic benefits. These traits are primarily regulated by numerous genes [8,9], such as growth hormone (GH), growth hormone-releasing hormone (GHRH), and insulin-like growth factors (IGFs), which have been identified as being associated with growth traits in many aquatic organisms [10,11]. It has been demonstrated that the knockout of myostatin (MSTN) can increase muscle mass [12]. Bone morphogenetic proteins (BMPs) have been proven to be important regulators of skeletal development and bone formation in multiple aquatic fish species [13]. Since growth traits are regulated by multiple genes, further exploration and identification of more candidate genes are needed to elucidate the genetic mechanisms underlying growth.

The application of second-generation sequencing technologies has made whole-genome resequencing widely used in the study of population structure and genetic variation, and it has been successfully applied to various valuable species [14]. Furthermore, through whole-genome selective sweep analysis, candidate genes associated with disease resistance [15], adaptability [16], and immune and productivity traits [17] have been identified. Genome-wide association study (GWAS) has emerged as a crucial method for investigating the correlation between genotypes and phenotypes, serving as a primary technology for identifying key loci associated with important traits through high-density single nucleotide polymorphisms (SNPs) [18]. It is widely applied in breeding improvement. The identification of candidate genes through significant SNP loci has been successfully applied in many species, including *Lateolabrax maculatus* [19], *Cyprinus carpio* [20], *Siniperca scherzeri* [21], *Trachinotus blochii* [22], and *Ictalurus punctatus* [23]. Through GWAS, many key candidate genes have been identified, which is of great significance for the study of growth and development in aquatic organisms.

*Procambarus clarkii* is becoming increasingly important in aquaculture. To accelerate research on the genetic improvement of growth, this study investigated the growth traits of 89 *P. clarkii* individuals from five cultured stocks across different regions in China using whole-genome resequencing technology. We aimed to identify single nucleotide polymorphisms (SNPs) and candidate genes associated with growth traits in *P. clarkii*. This study will provide valuable resources for further elucidating the genetic mechanisms of growth traits in *P. clarkii* and offer references for the future selective breeding of fast-growing strains.

## 2. Results

### 2.1. Resequencing Data and SNP Calling

Following whole-genome resequencing of the five cultured stocks, low-quality data were filtered using FASTP (v0.23.4). A total of 2644.85 Gb of clean data was obtained across all samples, with an average of 29.72 Gb per sample. The average Q30 value for the clean reads across all samples was 96.08%, and the GC content of the data was below 44%, indicating that the sequencing results were of high quality. The clean reads were mapped to the reference genome using BWA, with mapping rates ranging from 90.32% to 99.01%. The average sequencing coverage depth for the samples mapped to the reference genome was 10.86×. Single nucleotide polymorphism (SNP) calling was performed using GATK (v4.0), and the initially identified SNPs underwent additional filtering for subsequent GWAS analysis. After final filtering, a total of 14,046,984 SNPs and 2,160,652 indels were retained.

### 2.2. Population Phylogeny and Genetic Structure

**Figure 1:**
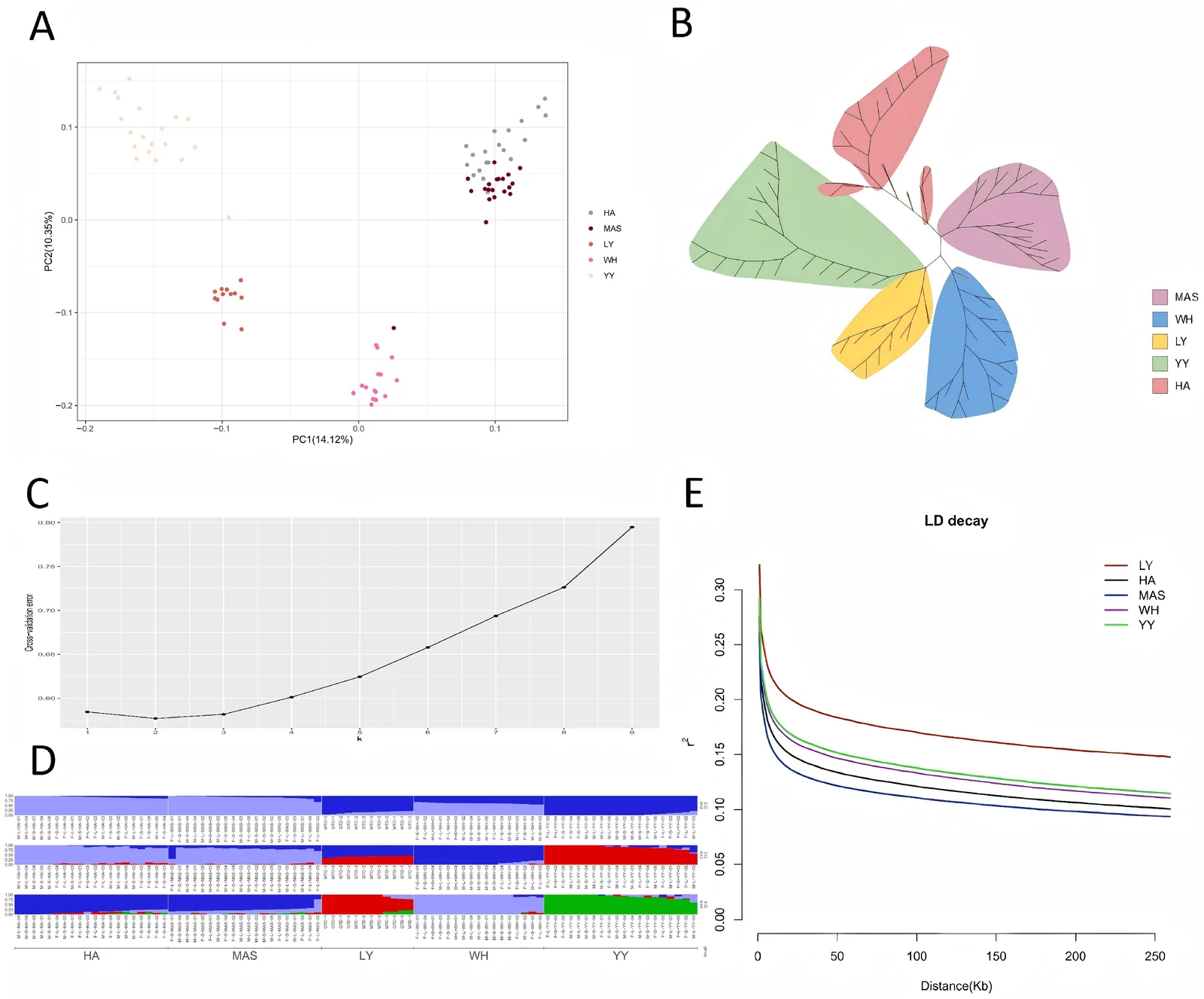
Analyses of population genetic structure of five cultured stocks of *P. clarkii*. (**A**) Population principal component analysis (PCA); (**B**) Phylogenetic tree; (**C**) Distribution of cross-validation (CV) error values corresponding to different K values; (**D**) Population genetic structure represented by different K values. Each column in the plot represents an individual; length of colored segments indicates proportion of ancestral genomic component. (**E**) Linkage disequilibrium (LD) decay plot.

To investigate the genetic structure of the five *P. clarkii* cultured stocks, we employed phylogenetic tree construction, population structure analysis, and principal component analysis (PCA) to visualize the genetic relationships among all 89 individuals. The PCA plot revealed distinct genetic separation among the five cultured stocks, with the 89 individuals clustering into five main groups corresponding to the Liuyang (LY), Yiyang (YY), Huai’an (HA), Wuhan (WH), and Ma’anshan (MAS) cultured stocks, respectively. Notably, the MAS and HA cultured stocks exhibited the closest genetic relationship (Figure 1A). The Neighbor-Joining (NJ) tree analysis (Figure 1B) corroborated the PCA results, showing that the different geographical cultured stocks formed five distinct evolutionary branches. Population structure analysis indicated that the optimal number of subpopulations (K) was 2, which yielded the minimum cross-validation error (Figure 1C,D). This genetic structure was consistent with the phylogenetic and PCA analyses, indicating that significant geographical genetic differentiation has occurred among *P. clarkii* cultured stocks.

The linkage disequilibrium (LD) decay patterns across these different *P. clarkii* cultured stocks were analyzed based on the correlation of allele frequencies between SNP pairs and their genomic distances. In this analysis, the LD decay was slowest in the LY cultured stocks, followed sequentially by the YY, WH, and HA cultured stocks, whereas the MAS cultured stocks exhibited the fastest LD decay (Figure 1E).

### 2.3. Historical Population Dynamics Analysis

**Figure 2:**
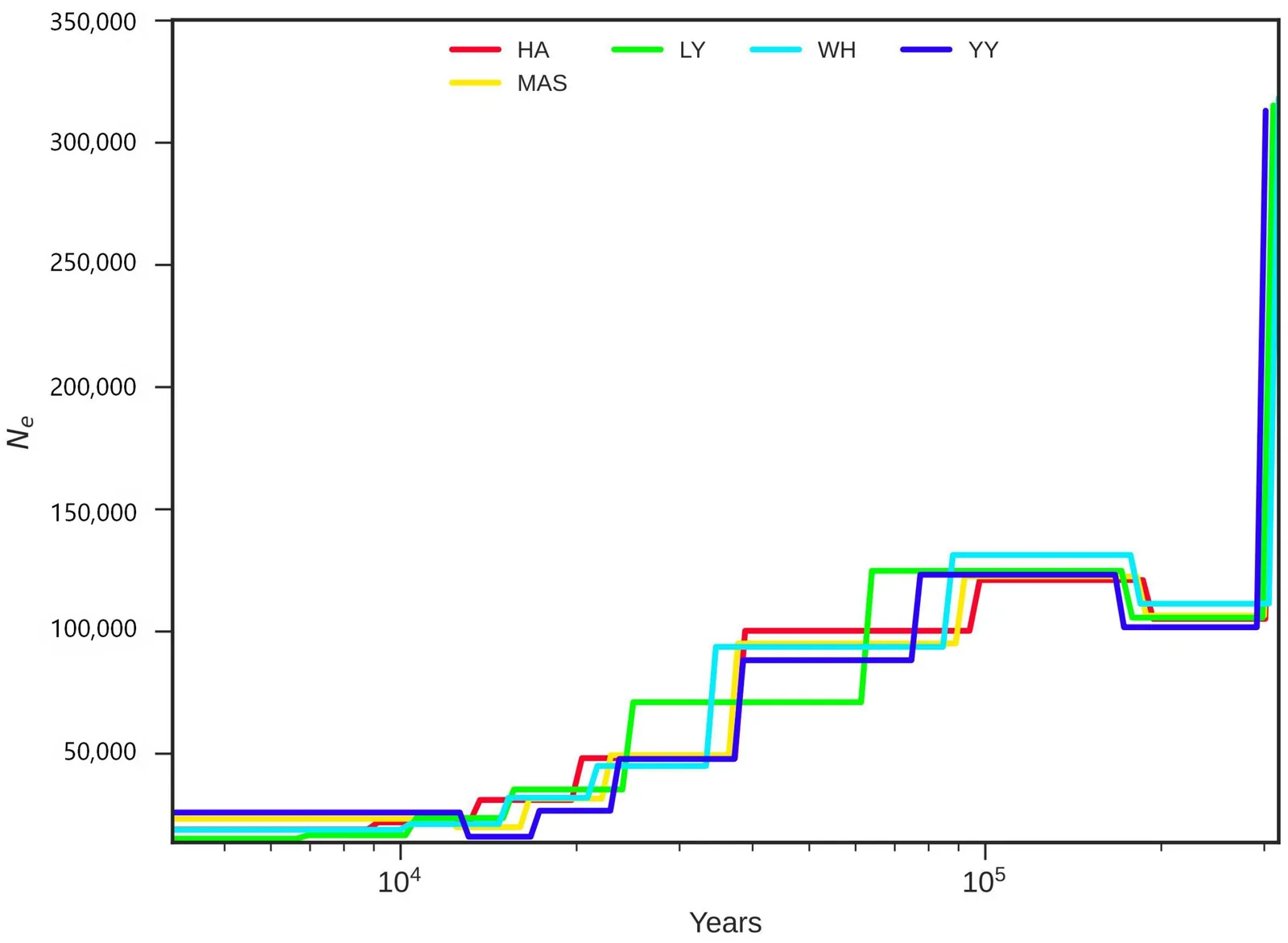
Historical population size dynamics of *P. clarkii*.

To investigate the genetic history of *P. clarkii* cultured stocks, we used SMC++ to estimate the effective population size across different historical periods. The results indicated that the *P. clarkii* cultured stocks experienced multiple bottleneck events (Figure 2), with its ancestors undergoing three distinct bottlenecks approximately 500,000, 100,000, and 40,000 years ago. These events are likely associated with glacial periods in North America and a major drought that occurred approximately 42,000 years ago [24,25]. The five cultured stocks exhibited similar historical dynamics, suggesting that they likely share a common ancestor and have been subjected to similar environmental conditions over a prolonged period.

### 2.4. Selective Sweep Analysis

**Figure 3:**
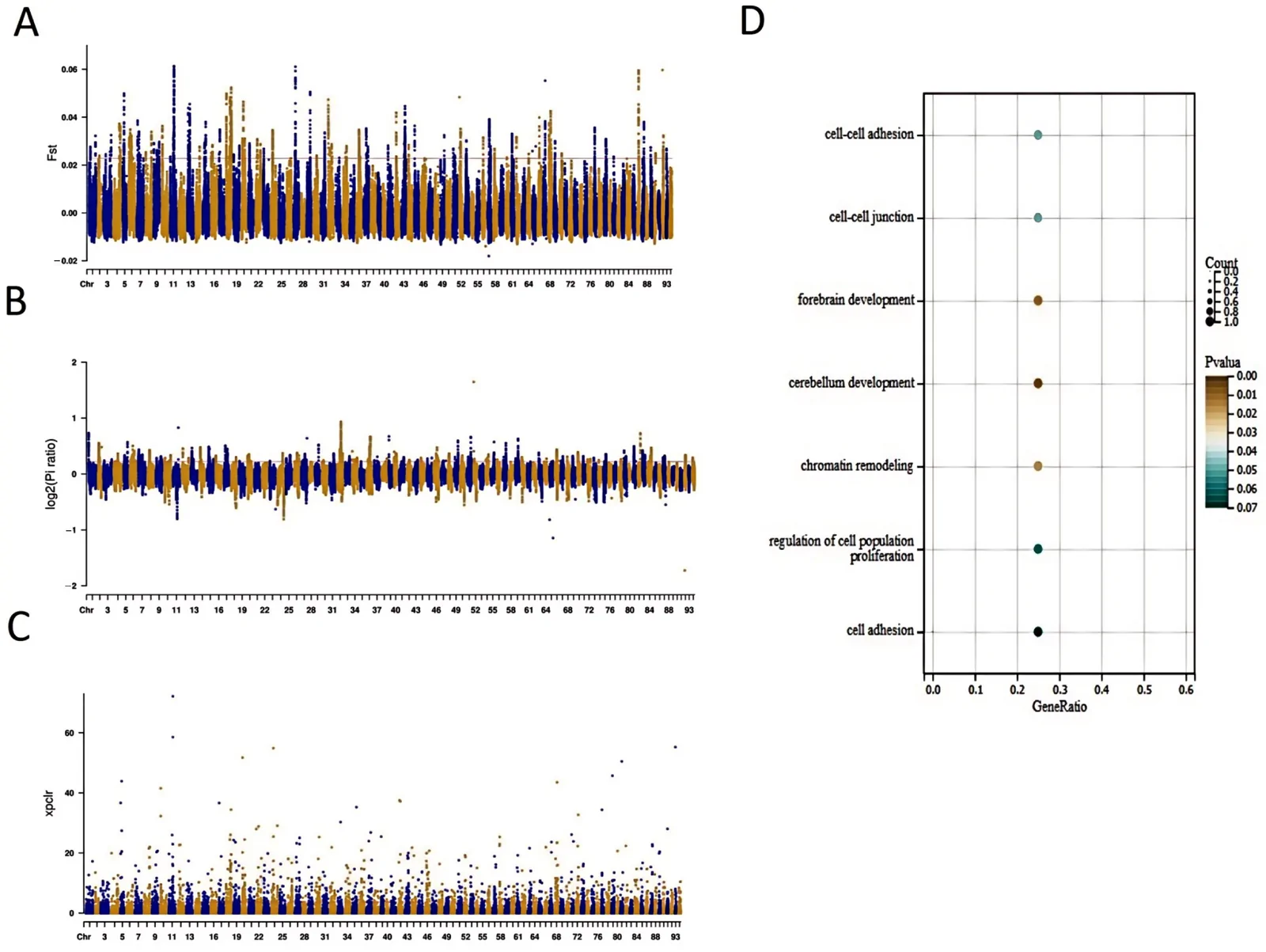
Identification of selective signals based on comparing large- and small-body-size cultured stocks. (**A**) Manhattan plot of signal selection using the Fst methods. (**B**) Manhattan plot of signal selection using the log_2_(Pi ratio) methods. (**C**) Manhattan plot of signal selection using the XP-CLR methods. Dashed lines represent the threshold of the top 1% values. The area above this line is regarded as a potential candidate region that may contain the selected feature. (**D**) Gene Ontology functional enrichment bubble plot of candidate growth-related genes.

To identify significant selective sweeps associated with growth traits in *P. clarkii*, we compared the genetic differences between the large- and small-body-size groups. This was accomplished using FST, Pi, and XP-CLR analyses (Figure 3A–C). Through these analyses, we identified 492, 622, and 2469 genes using the FST, Pi, and XP-CLR methods, respectively. Notably, we detected significant selective sweep signals associated with both previously characterized and newly identified functional genes. The key candidate genes identified at the intersection of the three selective sweep analyses include Nephrin, Somatostatin, zinc finger protein 154, and yeti. GO functional enrichment analysis (Figure 3D) was performed on these selected candidate genes. Both sets of candidate genes were significantly enriched in the following GO terms: “regulation of cell population proliferation” (GO:0042127), “forebrain development” (GO:0030900), “cerebellum development” (GO:0021549), “cell-cell adhesion” (GO:0098609), “chromatin remodeling” (GO:0006338), “regulation of cell shape” (GO:0008360), and “cell adhesion” (GO:0007155).

### 2.5. Genome-Wide Association Study (GWAS)

**Figure 4:**
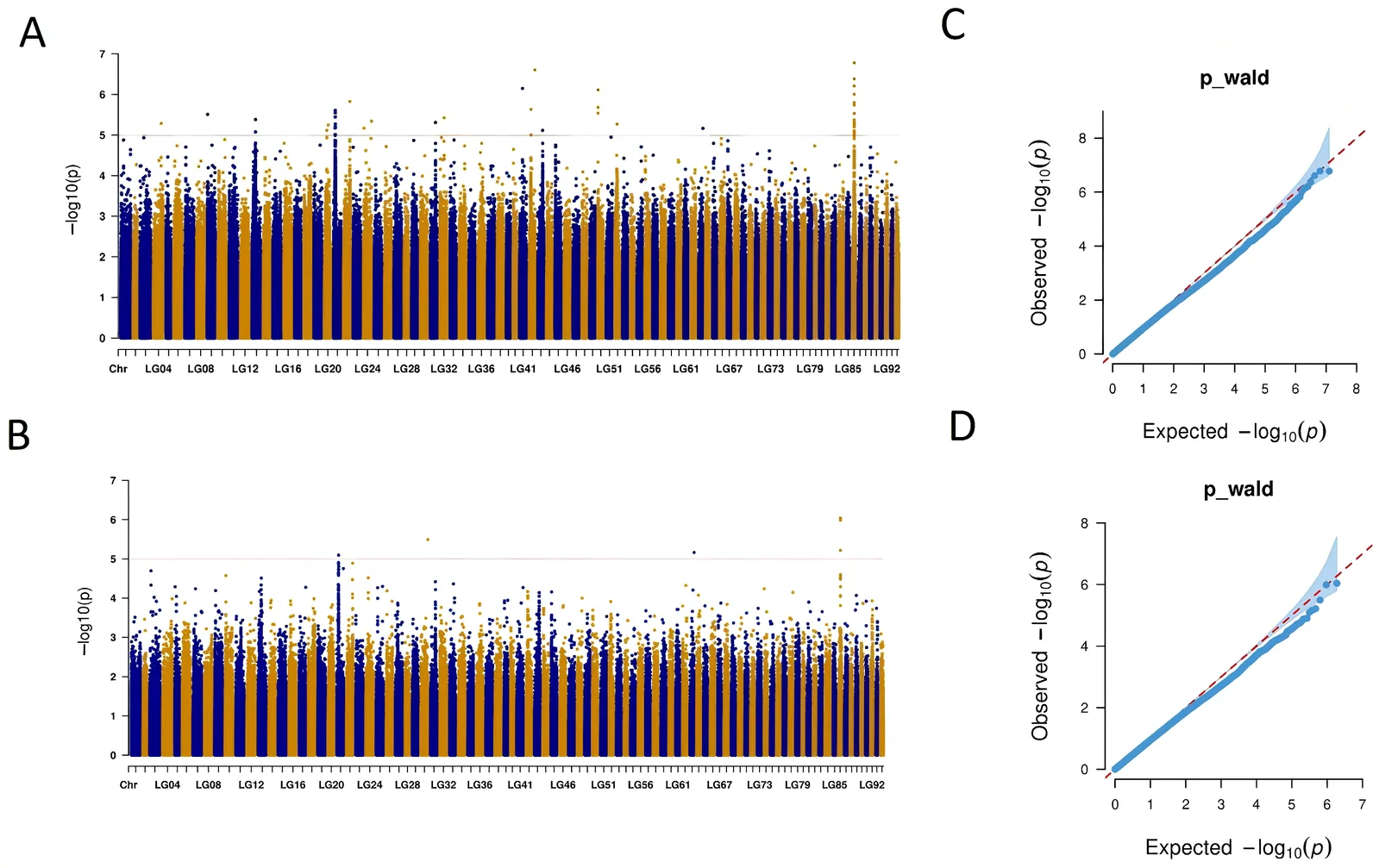
Genome-wide association analysis was conducted on the large- and small-body-size groups using SNP and InDel data. (**A**,**B**) The Manhattan diagram. (**C**,**D**) The quantile–quantile (Q–Q) diagram.

A genome-wide association study (GWAS) was conducted on 89 *P. clarkii* individuals. A total of 14,046,984 SNP loci were subjected to the GWAS using the GEMMA software (v0.98.5) for the combined large- and small-body-size groups of *P. clarkii*. Specifically, the kinship matrix among the samples was first calculated and subsequently used as a covariate in the association analysis. The GWAS results were visualized using Manhattan (Figure 4A,B) and quantile–quantile (Q-Q) plots (Figure 4C,D). Based on the whole-genome resequencing data from the 89 individuals, we investigated all mutation sites through GWAS and ultimately identified three major candidate genes associated with body size: nephrin, Cullin-associated and neddylation-dissociated protein 1 (CAND1), and BIRC8. Notably, nephrin overlaps with the significant GWAS signal peak, highlighting the importance of these genomic regions in shaping the growth trait.:

## 3. Discussion

In this study, we investigated *P. clarkii* from five cultured stocks in China (MAS, LY, YY, WH, and HA) through genetic evolutionary analysis, historical population dynamics analysis, selective sweep analysis, and a genome-wide association study (GWAS). The primary focus was on screening candidate genes associated with the key trait of growth using whole-genome resequencing technology.

We also characterized the genetic relationships and population structure of the five *P. clarkii* populations using a Neighbor-Joining (NJ) phylogenetic tree and principal component analysis (PCA). The phylogenetic tree clearly distinguished the five cultured stocks. Similarly, the PCA and population structure analyses revealed consistent population structuring. These results indicate that the five cultured stocks—MAS, LY, YY, WH, and HA—have undergone significant genetic differentiation. However, the MAS and HA cultured stocks exhibit a closer genetic relationship. This is likely due to prolonged artificial selection and increased gene flow facilitated by geographical proximity of these Chinese cultured stocks and their similar genetic backgrounds, which has led to genetic admixture and a lower level of genetic differentiation between these two cultured stocks.

In the genetic improvement of aquatic organisms, numerous studies have focused on growth-related traits [26,27,28,29,30]. In this study, to mitigate method-specific false positives in the selective sweep analyses, we took the intersection of three methods (FST, Pi, and XP-CLR). Nevertheless, we cannot completely exclude the possibility that residual population structure may have a marginal impact on extreme outliers. Using three selective sweep analysis methods based on FST, Pi, and XP-CLR, we identified 492, 622, and 2469 genes, respectively, with several growth-related genes being selected. By identifying the genes at the intersection of these three analyses, we ultimately determined the candidate genes within four candidate regions: Nephrin, Somatostatin, zinc finger protein 154, and yeti. This suggests that these candidate genes may be involved in growth determination. Nephrin has been shown to influence growth and development in Chinese Mitten Crab [31]. Somatostatin (SST) is a tetradecapeptide that was initially isolated from the sheep hypothalamus and characterized as a physiological inhibitor capable of suppressing the secretion of pituitary growth hormone (GH) [32]. SST has been shown to inhibit the mRNA expression of insulin-like growth factors 1 and 2 (IGF-1 and IGF-2) in the liver of the spotted scat, thereby influencing the regulation of growth in groupers [33]. SST can also regulate zebrafish growth; notably, zebrafish with an sst4 loss-of-function mutation generated via CRISPR/Cas9 technology exhibit significantly accelerated growth [34]. Members of the zinc finger protein (ZNF) family have been demonstrated to play critical roles in cell differentiation and growth [35]. ZNF154 may inhibit the Wnt/β-catenin signaling pathway by upregulating NLK, thereby suppressing cell proliferation and migration [36]. Research on the YETI protein has indeed provided the first experimental evidence indicating that BCNT family members are essential for normal growth, development, and individual survival [37,38].

To further investigate the potential roles of these candidate genes in body size traits, we conducted Gene Ontology (GO) enrichment analysis to gain an in-depth understanding of their functions, roles, and associated biological processes and pathways. These analytical tools are crucial for interpreting biological data and guiding future research. Among the candidate genes identified through selective sweep analysis, Nephrin overlapped with the significant GWAS signal peak. Furthermore, the GWAS identified two additional candidate genes: Cullin-associated and neddylation-dissociated protein 1 (CAND1) and BIRC8. CAND1 promotes cardiomyocyte proliferation and improves cardiac regeneration following cardiac injury by facilitating FBXW11-mediated K48-linked ubiquitination and degradation of Mob1b [39]. Additionally, CAND1 has been identified as an important regulator of adipogenesis [40]. BIRC8 (also known as ILP-2) belongs to the inhibitor of apoptosis protein (IAP) family and functions as an apoptosis inhibitor, potentially protecting cells from apoptotic stimuli and suppressing apoptosis [41,42]. In this study, whole-genome resequencing identified a total of seven candidate genes. These findings are crucial for understanding the growth and development of crustacean aquatic organisms and will provide valuable guidance for future practical applications.

In this study, we performed whole-genome resequencing on five distinct *P. clarkii* cultured stocks and jointly identified candidate growth-related genes using three selective sweep methods and a genome-wide association study (GWAS), further elucidating the genetic basis of growth in *P. clarkii*. Building on these findings, further exploration of the underlying mechanisms of these genes is warranted. These findings are not only of significant importance in the field of basic biology but also hold substantial value for aquaculture-related fields, including the study of growth mechanisms in *P. clarkii* and its genetic improvement through selective breeding. Additionally, whole-genome resequencing holds potential applications for investigating invasion routes and expansion ranges, as well as for assessing adaptive potential, thereby providing crucial information to inform targeted ecological management and control strategies.

## 4. Materials and Methods

### 4.1. Sample Sources and Whole-Genome Resequencing

A total of 89 *P. clarkii* individuals, comprising 45 large-bodied and 44 small-bodied individuals, were collected from the cooperative breeding farm of Hunan Normal University. The sample collection included 20 individuals from HA (10 large, 10 small), 20 from MAS (10 large, 10 small), 17 from WH (9 large, 8 small), 12 from LY (6 large, 6 small), and 20 from YY (10 large, 10 small). The average body length of a large body is 102.21 ± 4.32 mm, and that of a small body is 50.02 ± 2.14 mm. Individuals with a body length ranging from 95.0 to 110.0 mm were selected for the large-bodied group, and individuals with a body length ranging from 45.0 to 55.0 mm were selected for the small-bodied group. A statistical test (*t*-test) confirmed that there was a statistically significant difference in body size between the two groups (*p* < 0.001). All individuals were from the same age cohort and farming cycle and had reached sexual maturity [43]. Body length was measured using a vernier caliper as the total length from the tip of the rostrum to the posterior end of the telson. Genomic DNA was extracted from the muscle tissues of *P. clarkii* using a genomic DNA extraction kit (Qingdao, China). The integrity and quality of the extracted DNA were assessed using a NanoDrop 2000 spectrophotometer (Thermo Fisher Scientific, Waltham, MA, USA) and 1% agarose gel electrophoresis. Subsequently, high-throughput sequencing was performed on the Illumina NovaSeq 6000 platform (Illumina, San Diego, CA, USA). The raw reads were filtered using fastp v0.23.4. The quality control process involved filtering out reads where bases with a quality score of Q ≤ 20 accounted for over 50% of the total bases, removing read pairs and adapter fragments, and discarding reads with an excessive number of N bases (specifically, those containing more than 5 Ns). The high-quality reads were then mapped to the *P. clarkii* reference genome (assembly version: GCF_020424385.1) using BWA−MEM [44]. gVCF files were generated for each individual sample. Joint-calling was subsequently performed to obtain individual variant calls, and GATK (v4.0) [45] was utilized for variant detection in each sample, followed by the merging of the gVCF files across all samples using GATK. Finally, the SNPs and indels were filtered based on the following criteria: QD < 2.0 || FS > 200.0 || MQ < 40.0 || MQRankSum < −12.5 || ReadPosRankSum < −8.0. Following hard filtering of the SNP VCF, further quality control was performed using VCFtools (v 0.1.17). The filtering criteria included: mapping quality (MQ) ≥ 40, quality by depth (QD) ≥ 2, missing rate ≤ 0.1, MAF ≥ 0.05, retention of only biallelic SNPs, linkage disequilibrium (LD) pruning (−indep−pairwise 100 50 0.2), and Hardy–Weinberg equilibrium filtering (−HWE 0.001). After LD pruning (−indep−pairwise 100 50 0.2) and these quality control filters were applied, a total of 14,046,984 high-quality SNP loci were ultimately obtained. These SNPs were then used to construct the phylogenetic tree, perform principal component analysis (PCA), and conduct population structure analysis.

### 4.2. Phylogeny and Population Genetic Structure

We compared the linkage disequilibrium (LD) among the five cultured stocks. The LD decay of molecular markers among different *P. clarkii* populations was calculated using PopLDdecay (v3.40) based on the squared allele frequency correlation (r^2^) statistic of two loci with a minor allele frequency (MAF) greater than 0.01, with the maximum distance between two SNPs set to 5 kb [46]. Principal component analysis (PCA) was performed using Plink (v1.9) software, followed by the generation of a PCA scatter plot using R. In the scatter plot, each point represents a sample; the greater the distance between two points, the larger the genetic background difference between the two samples, and similar individuals are clustered into one group. Plink (v1.9) software was used to calculate the genetic distances between samples and construct the phylogenetic tree. Finally, the NJ tree was visualized via iTOL (v6.8) [47]. The population structure of all samples was analyzed using the Admixture (v1.3.0) software [48]. First, the accurate and appropriate number of subpopulations (K value) was determined. The K value was pre-set from 1 to 9, and the cross-validation error after assigning each sample to the K−th subpopulation was calculated.

### 4.3. Inference of Population History Using SMC++

Based on 14,046,984 SNP loci, SMC++, a population genetics method that infers population history from whole-genome sequence data, was employed to infer the population history of the *P. clarkii* population. Specifically, SMC++ (version: terhorst/smcpp:latest) [49] was used for the population history inference, with a generation time (g) of 1 and a per-generation nucleotide mutation rate of 4.59 × 10^−9^ [50].

### 4.4. Genome-Wide Selective Sweep Analysis

Based on 14,046,984 SNP loci, by comparing the allele frequencies between large- and small-bodied groups, three methods (FST, Pi, and XP-CLR) were used to conduct genome-wide selective sweep analysis to identify differences between these two body size groups of *P. clarkii*. Regarding the definitions, Pi is the expected heterozygosity per locus obtained from the average of difference sequences of all samples; Fst is the inbreeding coefficient of subpopulations, used to estimate the pairwise gene composition of candidate genes between paired subpopulations; Fst is a measure of population differentiation between groups, and the larger the differentiation index, the greater the difference [51]; XP-CLR, the cross-population composite likelihood ratio test, is a likelihood method based on selective sweep. XP-CLR utilizes multilocus allele frequency differences between the large- and small-bodied groups to establish a model. The Pi, Fst, and XP-CLR values were sorted from largest to smallest, and the top 1% were selected as the significant threshold line to determine whether they occurred in the studied groups, and to screen out the selected gene intervals. The windows identified jointly by these three methods are considered as putative selective sweeps, and the genes overlapping among the three methods were selected as candidate genes.

### 4.5. Genome-Wide Association Study

A genome-wide association study (GWAS) on the correlation between body size phenotypes and genotypes was conducted using the mixed linear model (MLM) in Genome-wide Efficient Mixed-Model Association (GEMMA) (V 0.98.5) [52]. QQ plots and Manhattan plots of −log10 (*p*-value) calculated from SNPs and InDels were generated using CMplot [53]. To ensure the reliability of the GWAS model analysis, a genomic inflation factor (λ) value close to 1.00 was used. The thresholds for genome-wide significance and suggestive association were set at 0.01/N and 0.05/N, respectively, where N represents the number of SNPs and InDels used in the GWAS analysis.

### 4.6. Functional Annotation of Candidate Genes

For the candidate genes, the coding protein sequences were extracted from the genomic CDS (Coding DNA Sequence) file. Gene function annotation relies on the previous gene structure prediction, where translated protein sequences are extracted from the genome and compared with mainstream databases to complete the functional annotation. The protein sequences of these candidate genes were aligned with the non-redundant protein sequences database (NR) using Diamond v0.8.22.84 software [54], with a cutoff e-value of 1 × 10^−5^. The extracted protein sequences were annotated using eggNOG-mapper to obtain gene symbols (Gene symbols). The online gProfiler tool [55] was then used to convert the gene symbols into Entrez Gene IDs. Finally, KOBAS 3.0 was used for GO functional enrichment analysis.

## Data Availability

The data from the study are available from the corresponding authors upon reasonable request.
